# TOE1 acts as a 3′ exonuclease for telomerase RNA and regulates telomere maintenance

**DOI:** 10.1093/nar/gky1019

**Published:** 2018-10-29

**Authors:** Tingting Deng, Yan Huang, Kai Weng, Song Lin, Yujing Li, Guang Shi, Yali Chen, Junjiu Huang, Dan Liu, Wenbin Ma, Zhou Songyang

**Affiliations:** 1 State Key Laboratory of Oncology in South China, Cancer Center, Collaborative Innovation Center for Cancer Medicine, MOE Key Laboratory of Gene Function and Regulation, School of Life Sciences, Sun Yat-sen University, Guangzhou 510006, China; 2 Guangzhou Regenerative Medicine and Health-Guangdong Laboratory (GRMH-GDL), Institute of Healthy Aging Research, Sun Yat-sen University, Guangzhou 510006, China; 3 Guangzhou Institute of Pediatrics, Guangzhou Women and Children's Medical Centre, Guangzhou 510623, China; 4 Verna and Marrs Mclean Department of Biochemistry and Molecular Biology, Baylor College of Medicine, One Baylor Plaza, Houston, TX 77030, USA

## Abstract

In human cells, telomeres are elongated by the telomerase complex that contains the reverse transcriptase hTERT and RNA template TERC/hTR. Poly(A)-specific ribonuclease (PARN) is known to trim hTR precursors by removing poly(A) tails. However, the precise mechanism of hTR 3′ maturation remains largely unknown. Target of Egr1 (TOE1) is an Asp-Glu-Asp-Asp (DEDD) domain containing deadenylase that is mutated in the human disease Pontocerebella Hypoplasia Type 7 (PCH7) and implicated in snRNA and hTR processing. We have previously found TOE1 to localize specifically in Cajal bodies, where telomerase RNP complex assembly takes place. In this study, we showed that TOE1 could interact with hTR and the telomerase complex. TOE1-deficient cells accumulated hTR precursors, including oligoadenylated and 3′-extended forms, which was accompanied by impaired telomerase activity and shortened telomeres. Telomerase activity in TOE1-deficient cells could be rescued by wild-type TOE1 but not the catalytically inactive mutant. Our results suggest that hTR 3′ end processing likely involves multiple exonucleases that work in parallel and/or sequentially, where TOE1 may function non-redundantly as a 3′-to-5′ exonuclease in conjunction with PARN. Our study highlights a mechanistic link between TOE1 mutation, improper hTR processing and telomere dysfunction in diseases such as PCH7.

## INTRODUCTION

Telomeres are long tandem repeats of 5′-TTAGGG-3′ at the ends of linear chromosomes that serve to protect the ends from untoward events such as recombination, degradation and end-to-end fusion, thereby ensuring genome integrity and stability (1,2). In human somatic cells, telomeres are estimated to shorten by 50–200 bp per cell cycle because of semi-conservative DNA synthesis (3,4). As telomeres become critically short, genomic instability and DNA damage response may ensue, which can lead to cancer, aging and other degenerative diseases.

Human telomeres are mostly maintained by the telomerase holoenzyme, the core of which comprises of the reverse transcriptase hTERT and the RNA template TERC/hTR (5–7). TERT expression is tightly controlled and usually low or undetectable in somatic cells, but elevated in germ cells, adult stem cells and highly proliferative cells such as lymphocytes (8–10). Myriad regulators ensure the proper maturation, assembly and telomeric targeting of the telomerase holoenzyme. Dysregulation in telomerase expression, processing, and/or activity can lead to dysfunctional telomeres and ultimately diseases such as dyskeratosis congenita (DC), a premature aging disorder. Prime examples can be found in DC patients harboring mutations in genes encoding a number of telomerase subunits and regulators. For instance, X-linked recessive DC has been linked to mutations in DKC1, autosomal dominant DC to TERT, hTR, RTEL1 and TIN2, and autosomal recessive DC to TERT, RTEL1, CTC1, NOP10, NHP2 and WRAP53/TCAB1 (11–14).

hTR is a non-coding RNA that serves as the template for telomere replication. It has a 3′ H/ACA snoRNA-like domain and shares structures such as the 5′ pseudoknot with lower organisms (15). Transcribed by RNA polymerase II (Pol II), hTR undergoes critical processing steps before maturing into the 451nt long, non-polyadenylated RNA species (15–17). Abnormal hTR expression and processing has been implicated in degenerative and malignant disorders. For example, reduced hTR levels are found in DC and idiopathic pulmonary fibrosis (IPF) patients carrying mutations of components of the H/ACA ribonucleoprotein complex, such as DKC1, NOP10 and NHP2 (18–22). More recently, mutations in the gene coding the poly(A)-specific ribonuclease (PARN), which impairs hTR 3′-end processing, were identified in IPF and DC patients (23–27). These findings further underline the importance of hTR processing to telomere maintenance and genome integrity.

Pontocerebellar hypoplasia (PCH) is a rare and highly heterogeneous group of disorders primarily characterized by early onset and hyperplasia of pons and cerebellum, with reported mutations in several genes including subunits of the tRNA splicing endonuclease complex (28). It is interesting to note that cerebellum hypoplasia also manifests in patients with telomere-related diseases such as Hoyeraal-Hreidarsson and Revesz syndromes (29–31). Of the 10 known PCH subtypes, PCH7 (MIM: 614969) appears associated with hypogonadism and was recently linked to mutations in the gene encoding TOE1 (target of Egr1, also known as hCaf1z) (28,32–33). TOE1 belongs to the DEDD family of deadenylases, although its physiological substrates remain largely unknown. In a genome-wide microscopy-based localization study (34), we and colleagues identified TOE1 as a protein specifically localized to Cajal bodies (CBs), which are sites of maturation for small nuclear RNAs (snRNAs) as well as hTR (35–40). Cells from PCH7 patients with TOE1 mutation displayed an accumulation of immature snRNAs, supporting a role of TOE1 in mediating snRNA maturation (33,41).

In this study, we tested the hypothesis that TOE1 might participate in hTR processing and contribute to telomere maintenance. We demonstrated that TOE1 could associate with telomerase in a DEDD domain-dependent manner. TOE1 deficiency (through RNAi and CRISPR/Cas9-mediated knockout) resulted in the accumulation of 3′-extended and oligoadenylated hTR precursors without decreasing the total hTR levels, which was accompanied by impaired telomerase activity and shortened telomeres in these cells. Diminished telomerase activity in these cells could be rescued by wild-type TOE1 but not by the deadenylase-dead mutant. Furthermore, *in vitro* analysis further supported TOE1 as an exonuclease directly targeting the 3′ end of hTR. TOE1 may function non-redundantly with PARN, to precisely trim hTR to its mature and functional form in CB. Our data combined reveal for the first time how TOE1 can facilitate telomere maintenance by regulating hTR biogenesis, and shed light on possible new mechanisms of the development and progression of telomere-related diseases such as PCH7, IPF and DC.

## MATERIALS AND METHODS

### Vectors, cell lines and RNAi

Full-length TOE1 (from the human ORFeome v5.1 library) (34) and TOE1 mutants including DEDD domain deletion (TOE1-ΔDEDD) and catalytically inactive TOE1 (D64A/E66A) were cloned into pLenti-FLAG-HA lentiviral vectors for N-terminal FLAG and HA tagging. For GST-tagging TOE1, pDEST-27 (Invitrogen) vector was used. cDNAs for DKC1, TCAB1, TERT and GFP were cloned into pLenti-FLAG-HA vectors for N-terminal FLAG and HA tagging as well. Full-length and mutant TOE1 were also cloned into the pET vector for tagging with the His tag, expressed in bacteria and purified with Ni sepharose 6 Fast Flow beads (GE healthcare, 17-5318-01).

HeLa and 293T cells were cultured in DMEM media supplemented with 10% fetal bovine serum and 1% penicillin-streptomycin. Immunostaining experiments were performed in HeLa cells while other transfection related experiments were done in 293T cells due to its high transfection efficiency. siRNA oligos were ordered from RiboBio and transfected into cells using Lipofectamine® RNAiMAX (Thermo Fisher Scientific). siTOE1-A: 5′-ATAGCATCAAGCCTGAAGA-3′; siTOE1-B: 5′-TACCCTGGAGTTCTGCAAC-3′; siTOE1-C: 5′-GCTCAAGTGTTCAATCTCA-3′; siPARN: 5′- GGAAGAAGAAAGACAGTTATT-3′. The RNAi negative control (siNC) was also purchased from RiboBio.

### Generation of TOE1 CRISPR/Cas9 KO cells

To generate inducible TOE1 KO cells, sgRNAs targeting the TOE1 locus were first cloned into the Lenti gRNA Blasticidin/Hygromycin-Lenti-Inducible-Cas9-neo vector described by Zhang Laboratory (Addgene) (42) for lentiviral packaging and infection of HeLa and 293T cells expressing tetracycline-inducible Cas9. sgRNAs targeting GFP were also generated as control. Two inducible lines were generated, using two different combinations of TOE1 gRNAs. To induce KO, 1 μg/ml doxycycline was added to cells for seven days. Successful KO was confirmed by western blot. After successful KO, the KO1 populations of cells were plated into 96-well plates to isolate single clones of TOE1 KO cells. To generate rescue cells, 293T cells were infected with lentiviruses encoding the corresponding genes and then selected with puromycin (1 μg/ml for 4 days). gRNAs sequences are:

TOE1-sgRNA1: 5′-AACTGCGCCATCGTCACTGT-3′;
TOE1-sgRNA2: 5′ -GCAACAACTTCAAGGAGATG-3′;
TOE1-sgRNA3: 5′- GAAGTTGTCACAGATGCTGG-3′;
GFP-sgRNA1: 5′-GAGCTGGACGGCGACGTAAA-3′;
GFP-sgRNA2: 5′- CAAGTTCAGCGTGTCCGGCG-3′.

### qRT-PCR, Q-TRAP, IP-TRAP and RNA co-immunoprecipitation (RIP)

qRT-PCR was carried out as described previously (43). Briefly, total RNA was extracted by TRIZOL (Thermo Fisher Scientific) and quantificated by Nanodrop 1000 (Thermo Fisher Scientific). A total of 1 μg RNA were treated with the RNase-free RQ DNase I (Promega) for 10 min at 37°C and then reverse transcribed using the RevertAid First Strand cDNA Synthesis kit (Thermo Fisher Scientific, #K1622), with hexamer or oligo(dT)_18_ primers. Quantitative polymerase chain reaction (qPCR) was carried out in Applied Biosystems StepOne™ Real-Time PCR Systems using the Power SYBR Green PCR Master Mix (Promega GoTaq Qpcr Master, A6002). Briefly, 10 μl GoTaq qPCR Master Mix, 0.2 μl upstream and downstream PCR primers (10 μM each), 1 μl cDNA and 8.8 μl H_2_O were mixed in each 20 μl reaction. The amplification program is: 95°C for 10 min, then 40 cycles of 95°C for 15 s, 60°C for 1 min. Melting curve program is: 95°C for 15 s, 60°C for 1 min, temperature increment of 0.3°C, 95°C for 15 s. Quantification was performed by comparative CT (ΔΔCT). The primers used were:

hTR FP: 5′-GGGAGGGGTGGTGGCCATTTTT-3′;
hTR RP: 5′-GAACGGGCCAGCAGCTGACATT-3′;
GAPDH FP: 5′-ACAACTTTGGTATCGTGGAAGG-3′;
GAPDH RP: 5′-GCCATCACGCCACAGTTTC-3′.

Real-time quantitative PCR-based Telomerase Repeated Amplification Protocol (Q-TRAP) analysis was performed as described previously (44). Immunoprecipitation followed by TRAP assays (IP-TRAP) was carried out as described previously (45). Briefly, cell lysates were incubated with anti-FLAG M2 beads (Sigma, A2220) at 4°C for 2 h and the eluates were used for Q-TRAP.

RNA immunoprecipitation (RIP) assays were carried out as described previously (24). Briefly, cell extracts were incubated with anti-FLAG M2 beads (Sigma, A2220) and subsequently washed five times with RIP buffer. Then RNA was extracted using the TRIzol reagent (Thermo Fisher Scientific). The extracted RNA was then analyzed by real-time quantitative PCR using the Power SYBR Green PCR Master Mix (Promega GoTaq Qpcr Master, A6002).

### *In vitro* deadenylation assays

For substrate preparation, full-length hTR cDNA was cloned into pGEM vector and the hTR-pGEM plasmids were used as PCR template to amplify the T7-hTR-459-7A linear cDNA, subsequently *in vitro* transcribed hTR-459–7A was obtained using the T7 Transcription Kit (Thermo Fisher Scientific, EP0111). Deadenylation assays were performed as described previously (46). Briefly, hTR-459-7A RNA substrate was incubated with 30 nM bacterially purified proteins at 37°C for 60 min. After incubation, RNA was extracted using RNeasy Micro Kit (QIAGEN, 74004) to perform the following 3′ RACE experiments. The primers used were: hTR-459-7A-T7-FP: 5′-TAATACGACTCACTATAGGGTTGCGGAGGGTGGGCCTGGGAGGGGTGGTGGCCATTT-3′; hTR-459-7A-RP: 5′-TTTTTTTAGCGAACTGCATGTGTGAGCCGAGTCCTGGGTGCACGT-3′.

### Analyzing hTR 3′ end using 3′ RACE

Preparation of hTR-specific libraries was based on the RNA ligase-mediated 3′ RACE approach previously described (23). RT-PCR amplification was carried out using the following primers.

Universal RT primer R1: 5′- CTACGTAACGATTGATGGTGCCTACAG; hTR-F1: 5′-GGGAGGGGTGGTGGCCATTTTT-3′; hTR-F2: 5′-CTCTGTCAGCCGCGGGTCTCTC-3′.

3′ RACE products were prepared for deep sequencing using the TruSeq Nano DNA LT Library Prep kit (Illumina). The raw data were paired end reads of 250 bp in length. Adaptor sequences were trimmed using Cutadapt (v1.14), and low-complexity or low-quality sequences were masked. The remaining reads were mapped to the hTR gene sequence and its surrounding 1 kb region from hg19 using the MEM algorithm of bwa v0.7.10-r789 with default parameters. Reads that mapped perfectly to hTR at the first base pair and the following 18 bp with ≤1 mismatch and no insertion/deletion were identified using the pairwise alignment algorithm from the R package Biostrings. Numbers of left-end reads that mapped to different positions at or around the 3′ end of hTR were counted, and their percentages in the total number of fragments enclosing the 3′ end of hTR were calculated. The original data have been submitted to SRA database with accession number SRP145157.

### Co-immunoprecipitation (Co-IP), western blotting and immunofluorescence (IF)

Co-immunoprecipitation (Co-IP), western blotting and IF were carried out as previously described (47). The antibodies used for IP and western are: mouse monoclonal anti-FLAG (Abmart, M20008), rabbit polyclonal anti-GST (CST, 2622S), rabbit polyclonal anti-GAPDH (Abmart, P30008), mouse monoclonal anti-Coilin (Abcam, ab87913), rabbit polyclonal anti-TOE1 (BETHYL, A303-643A), rabbit polyclonal anti-DKC1 (Santa Cruz, sc-48794), anti-FLAG M2 Affinity Gel (Sigma, A220).

Primary antibodies for IF are: mouse monoclonal anti-Coilin (Abcam, ab87913), rabbit polyclonal anti-TOE1 (BETHYL, A303-643A), rabbit polyclonal anti-DKC1 (Santa Cruz, sc-48794), mouse polyclonal anti-DKC1 (Santa Cruz, sc-373956), rabbit polyclonal anti-Coilin (Santa Cruz, H-300). Secondary antibodies include DyLight488 goat anti-rabbit IgG (Liankebio, LK-GAR4882), Dylight488 goat anti-mouse IgG (Liankebio, LK-GAR4881), Alexa Fluor^®^ 555 Donkey Anti-Mouse IgG (Thermo Fisher Scientific, A-31570), Alexa Fluor^®^ 555 Donkey Anti-Rabbit IgG (Thermo Fisher Scientific, A-31572).

### Terminal restriction fragment (TRF) analysis

Average length of telomeres was determined using the terminal restriction fragment (TRF) assay as described previously with modifications (48). Isolated genomic DNA was digested with Rsa I and Hinf I before being resolved by electrophoresis (0.7% agarose gel), denatured and hybridized with a radiolabeled (TTAGGG)_4_ probe. Signal intensity was quantified on a PhosphorImager. Average telomere length was analyzed and calculated using the ImageJ software.

### *In vitro* telomerase reconstruction assays

hTERT cloned in T7 promoter-driven phTERT-HA2 vector are gifts from Dr Jiunn-Liang Chen. *In vitro* translation was performed by using the TNT kit (Promega, L4610). cDNA of full-length hTR was cloned into pGEM vector. hTR variant templates were PCR-amplified from the hTR-pGEM plasmids by using oligonucleotides that contain a T7 promoter sequences in the forward primer. hTR variants were generated by using the T7 Transcription Kit (Thermo, EP0111). *In vitro* translated hTERT products were 1:10 diluted with telomerase reconstruction buffer (25 mM Tris–HCl pH 7.4, 2.6 mM KCl, 1 mM MgCl_2_, 136 mM NaCl, 1 mM EGTA, 10% glycerol, 1 mM DTT, 1× proteinase inhibitor cocktail (Sigma) and 0.5 U/μl of RNasin Ribonuclease Inhibitor (Promega) and incubated with hTR (final concentration of 10 nM) at 37°C for 30 min. Telomerase activity was measured by Q-TRAP. The primers used were:

hTR-T7-FP: 5′-TAATACGACTCACTATAGGGTTGCGGAGGGTGGGCCTGGGAGG
GGTGGTGGCCATTT-3′;
hTR-451 RP: 5′-GCATGTGTGAGCCGAGTCCTGGGTGCACGT-3′;
hTR-452 RP: 5′-TGCATGTGTGAGCCGAGTCCTGGGTGCACG-3′;
hTR-453 RP: 5′-CTGCATGTGTGAGCCGAGTCCTGGGTGCAC-3′;
hTR-454 RP: 5′-ACTGCATGTGTGAGCCGAGTCCTGGGTGCA-3′;
hTR-455 RP: 5′-AACTGCATGTGTGAGCCGAGTCCTGGGTGC-3′;
hTR-459 RP: 5′-AGCGAACTGCATGTGTGAGCCGAGTCCTGG-3′;
hTR-451-7A-RP: 5′-TTTTTTTGCATGTGTGAGCCGAGTCCTGGGTGCACGT-3′.

## RESULTS

### TOE1 associates with telomerase subunits

**Figure 1.**
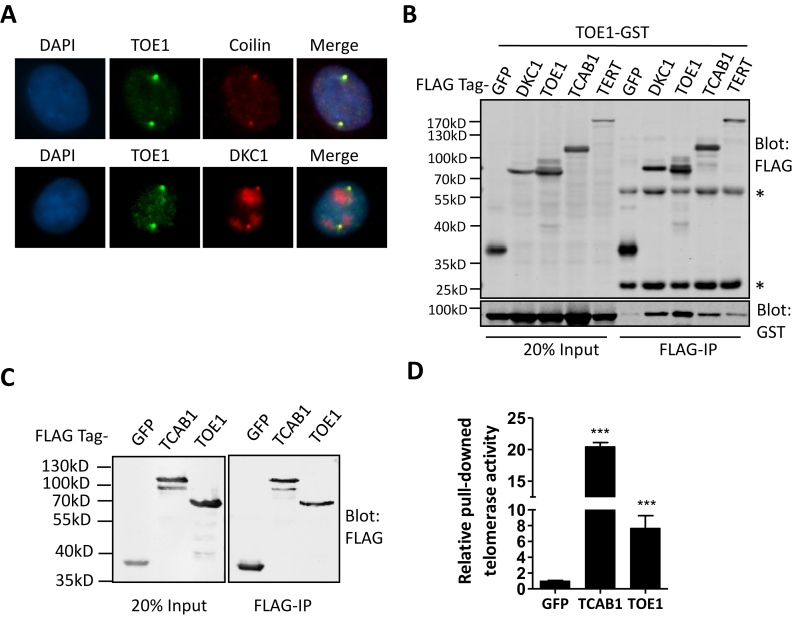
CB-localized TOE1 is a telomerase-associated protein. (**A**) The co-localization of endogenous TOE1 (green) with endogenous Coilin or DKC1 (red) in HeLa cells was assessed by IF. DAPI was used to stain the nuclei. (**B**) GST-tagged TOE1 was transiently co-expressed in 293T cells with FLAG-HA-tagged GFP, DKC1, TOE1, TCAB1 or TERT. Co-IP was done using anti-FLAG agarose beads. The immunoprecipitates (IP) were blotted with anti-FLAG and GST antibodies. FLAG-HA-GFP served as a negative control. * indicates positions of denatured heavy and light chains. (**C**) 293T cells transiently expressing FLAG-HA tagged GFP, TCAB1 or TOE1 were harvested for IP using anti-FLAG agarose beads. The IP were blotted with anti-FLAG antibodies. (**D**) The relative telomerase activity of IP from (C) was quantified by the Q-TRAP assay. A set of representative data from three biological replicates are shown here. Error bars represent s.d. (*n* = 3, technical replicates), *** *P* < 0.001, one-tailed unpaired *t*-test.

In previous immunoprecipitation-mass spectrometry (IP-MS) studies from our lab and others, several known telomerase subunits and telomerase-associated proteins, including DKC1, GAR1, NHP2 and NOP10 were found in the TOE1 complex (33,34), suggesting possible association of TOE1 with the telomerase holoenzyme. Consistent with these findings, immunofluorescence (IF) analysis in HeLa cells indicated co-staining of endogenous TOE1 and DKC1, the telomerase subunit that binds to hTR’s H/ACA hairpin (Figure 1A). Next, we determined whether TOE1 could bind to telomerase subunits by Co-IP using cells ectopically expressing GST-tagged TOE1 and FLAG-tagged telomerase subunits. In line with our IP-MS and IF data, GST-TOE1 was able to co-immunoprecipitate with FLAG-DKC1 (Figure 1B). Notably, GST-TOE1 could also be pulled down by itself as well as FLAG-tagged TERT and TCAB1 (Figure 1B). More importantly, exogenously expressed FLAG-tagged TOE1 could bring down significant amount of active telomerase in 293T cells as measured by Q-TRAP assays (Figure 1C and D). The level of telomerase activity brought down by TOE1 was ∼1/3 of that brought down by FLAG-TCAB1, suggesting that TOE1 may not be a core subunit of the telomerase holoenzyme.

### TOE1 domains are critical for its localization to Cajal bodies and interaction with telomerase

**Figure 2.**
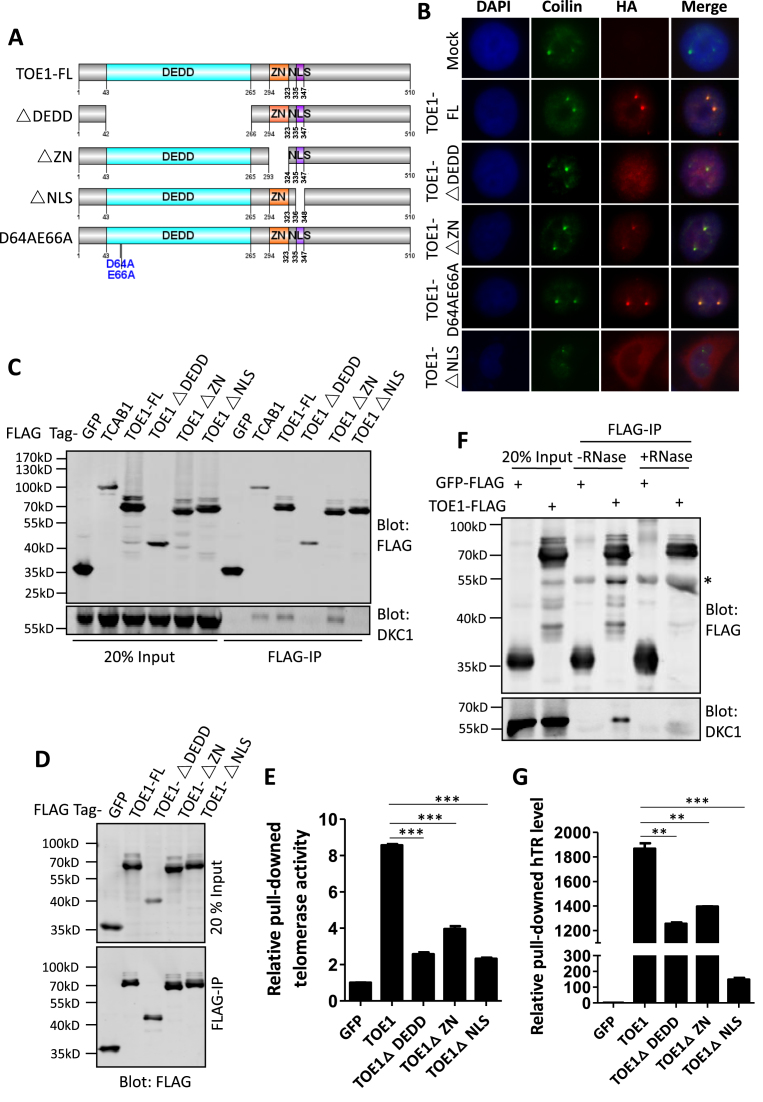
The TOE1 domains are critical for its localization and telomerase interaction. (**A**) Schematic representation of the human TOE1 protein. DEDD, deadenylation domain. ZN, zinc finger domain. NLS, nuclear localization signal. D64 and E66 are two conserved acidic residues predicted to play a role in catalysis, and were mutated to Ala (TOE1-D64AE66A). TOE1-FL, full-length wild-type TOE1. TOE1ΔDEDD, DEDD domain deletion mutant. TOE1ΔZN, ZN deletion mutant. TOE1ΔNLS, NLS deletion mutant. (**B**) HeLa cells transiently expressing FLAG-HA-tagged wild-type or mutant TOE1 (HA-red) were analyzed by IF using antibodies against HA and endogenous Coilin (green). DAPI was used to stain nuclei. (**C**) 293T cells transiently expressing FLAG-HA-tagged wild-type and mutant TOE1 were harvested for immunoprecipitation using anti-FLAG agarose beads. FLAG-HA- TCAB1 and FLAG-HA-GFP served as positive and negative controls, respectively. The IP were blotted with antibodies against FLAG and DKC1. (**D**) Western blot analysis of whole-cell extract (Input) and FLAG IP from 293T cells transiently expressing FLAG-HA-tagged GFP, TOE1-FL, TOE1ΔDEDD, TOE1ΔZN and TOE1ΔNLS using an anti-FLAG antibody. (**E**) The relative telomerase activity of IP from (D) was quantified by Q-TRAP. A set of representative data from three biological replicates are shown here. Error bars represent s.d. (*n* = 3, technical replicates). **P < 0.01, ***P < 0.001, one-tailed unpaired *t*-test. (**F**) 293T cells transiently expressing FLAG-HA-tagged GFP or TOE1 were immunoprecipitated in the presence or absence of RNase using an anti-FLAG antibody. Whole-cell extract (Input) and the IP were western blotted as indicated. * indicates the positions of denatured heavy chains. (**G**) For RIP assays, RNA was extracted from anti-FLAG IP from 293T cells transiently expressing wild-type or mutant TOE1. FLAG-HA-GFP served as a negative control. A set of representative data from three biological replicates are shown here. Error bars represent s.d. (*n* = 3, technical replicates), **P < 0.01, ***P < 0.001, one-tailed unpaired *t*-test.

TOE1 contains a C3H-type zinc finger (ZN), a nuclear localization signal (NLS) peptide and a DEDD domain with deadenylase activity (Figure 2A). Two conserved acidic residues (D64 and E66) within the DEDD domain are thought to be critical for TOE1-mediated deadenylation (49–51,46). Deleting the DEDD domain, but not ZN, not only prevented TOE1 from localizing to CBs (Figure 2B), but also abrogated its interaction with DKC1 (Figure 2C). Furthermore, TOE1 ΔDEDD also brought down less active telomerase in IP-TRAP assays (Figure 2D and E), supporting the importance of its deadenylase activity to TOE1 function. Not surprisingly, deletion of the NLS, which should block CB localization of TOE1, disrupted TOE1 binding to DKC1 and active telomerase (Figure 2B–E). Interestingly, ZN deletion also diminished TOE1’s ability to bring down telomerase activity, despite its lack of effect on TOE1 localization or DKC1 association (Figure 2B–E). The exact role of ZN domains is unclear, although their presence in proteins that interact with nucleic acids hints at a possible involvement in RNA binding. Our data here suggest that nucleic acid binding might be required for TOE1 association with telomerase, especially given its CB localization and known activity in modulating snRNA maturation (33,34). Indeed, when co-IP of DKC1 and TOE1 was performed in the presence of RNase, TOE1-DKC1 association was abolished (Figure 2F and Supplementary Figure S1), supporting RNA-dependent interaction between TOE1 and DKC1. We reasoned that TOE1 might localize to CBs to deadenylate RNAs, with hTR being one of its substrates and bridging its binding to telomerase. To test this idea, we carried out RNA immunoprecipitation (RIP) experiments using cells exogenously expressing various TOE1 mutants. As shown in Figure 2G, deleting any of the main domains, NLS, ZN or DEDD, drastically reduced the amount of hTR brought down by TOE1, further confirming the importance of CB localization, nucleic acid binding and deadenylase activity to TOE1–telomerase interaction.

### TOE1 is important for hTR 3′ maturation and telomere length maintenance

**Figure 3.**
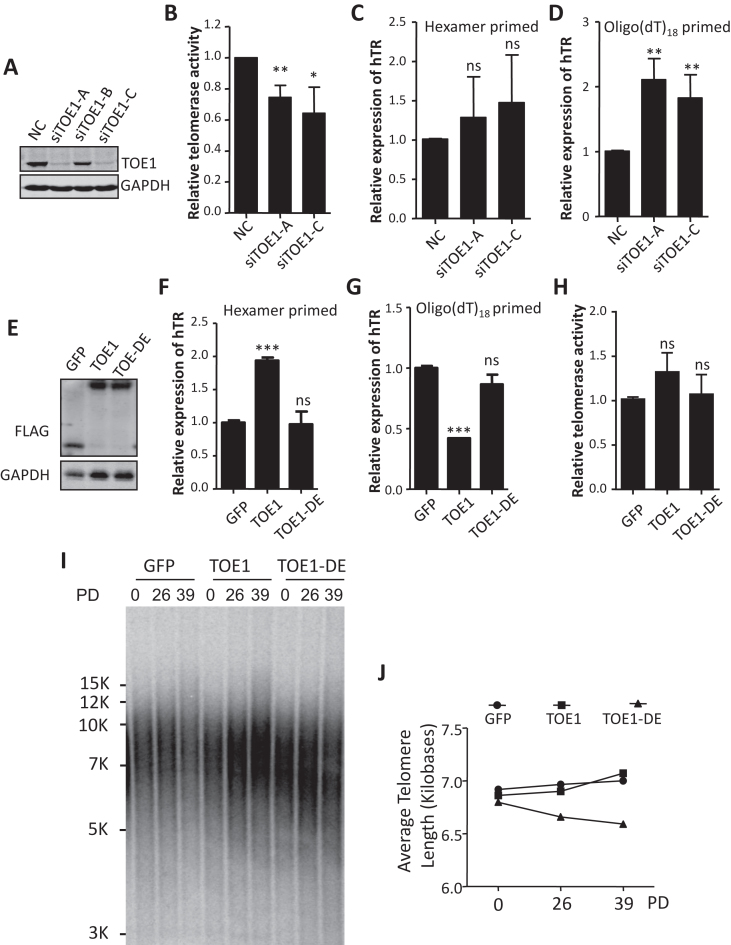
TOE1 is important for hTR processing and telomere maintenance. (**A**) Three siRNAs targeting TOE1 were transiently transfected into HeLa cells. A non-targeting siRNA scramble served as a negative control (NC). At 48 h after transfection, knockdown efficiency was assessed by western blot using antibodies against TOE1 and GAPDH. (**B**) The relative telomerase activity of cells from (A) was quantified by Q-TRAP assay (*n* = 3, biological replicates). Error bars represent s.d., *P < 0.05, ** *P* < 0.01, one-tailed unpaired *t*-test. (**C** and **D**) RNA was extracted from cells from (A) for qRT-PCR analysis of hTR using hexamer (C) or oligo(dT) (D) primers. Error bars represent s.d. (*n* = 3, biological replicates). ** *P* < 0.01, one-tailed unpaired *t*-test. ns, not significant. (**E**) Western blot analysis of whole-cell extracts from 293T cells that stably expressed FLAG-HA-tagged GFP, TOE1-FL and TOE1-DE. (**F** and **G**) RNA was extracted from cells from (E) for qRT-PCR analysis of hTR using hexamer (F) or oligo(dT) (G) primers. Error bars represent s.d. (*n* = 3, technical replicates). *** *P* < 0.001, one-tailed unpaired *t*-test. ns, not significant. (**H**) The relative telomerase activity of cells from (E) was quantified by Q-TRAP. A set of representative data from three biological replicates are shown here. Error bars represent s.d. (*n* = 3, technical replicates). Significance was calculated using one-tailed unpaired *t*-test. ns, not significant. (**I**) 293T cells that stably expressed FLAG-HA-tagged GFP, TOE1-FL and TOE1-DE were generated and passaged over time. Cells were harvested at the indicated time points for terminal restriction fragment (TRF) analysis to determine average telomere length. PD, population doubling. (**J**) Quantification of data from (I) using the ImageJ software.

Transcribed from its own promoter by RNA polymerase II, fully mature hTR (451 nt) lacks a poly(A) tail (52). Its 3′ end is processed through exonucleolytic cleavage up to the region bound by H/ACA proteins (53). The widely expressed cap-dependent poly(A) deadenylase PARN has been reported as required for removing the oligo(A) tail from nascent hTR (24,54). Depleting PARN has been shown to reduce the amount of mature hTR while increasing the proportion of oligo(A)-containing hTR, resulting in decreased telomerase activity and shortened telomeres (23). PARN contains a DEDD domain in addition to RRM and R3H RNA-binding domains (50,55–56), and is a member of the CAF1 family of ribonucleases, to which TOE1 also belongs. Our data thus far suggest that TOE1 might participate in hTR processing in CB. Consistent to our hypothesis, TOE1 was recently found to play a redundant role with PARN in hTR biogenesis, as knocking down TOE1 alone could not notably affect telomerase activity (41). Here, we assessed telomerase activities and hTR levels in HeLa cells RNAi depleted of TOE1 by multiple oligos. Two siRNA oligos appeared effective in knocking down TOE1 expression (Figure 3A, siTOE1 A and C). Compared to control cells, TOE1-depleted cells exhibited a moderate but reproducible reduction in telomerase activity by Q-TRAP assays (Figure 3B), with no decrease in total hTR levels as determined by hexamer-primed RT-qPCR (Figure 3C). Similar to what was observed in cells depleted of PARN (41), the level of oligo(A)-containing hTR also increased upon TOE1 knockdown (KD) as measured by oligo(dT)-primed RT-qPCR (Figure 3D).

Since our HeLa cells have relatively short telomeres, we next examined TOE1 activity using 293T cells stably overexpressing TOE1. We found adenylated forms of hTR were significantly lowered in TOE1-overexpressing cells compared to control cells or those expressing the catalytically inactive point mutant TOE1-D64A/E66A (TOE1-DE) (Figure 3E–G), although the telomerase activity or telomere length in TOE1 cells was mostly unaffected (Figure 3H–J). Ectopic expression of TOE1-DE did lead to a moderate but reproducible decrease in telomere length (Figure 3I and J; Supplementary Figure S2), without any apparent accumulation of oligoadenylated hTR or changes in total hTR levels (Figure 3F and G), suggesting that the catalytically inactive TOE1-DE might have acted as a dominant negative. Taken together, these results suggest an important role for TOE1 in hTR maturation and telomere length maintenance.

### TOE1 is required for telomerase activity and telomere length maintenance

**Figure 4.**
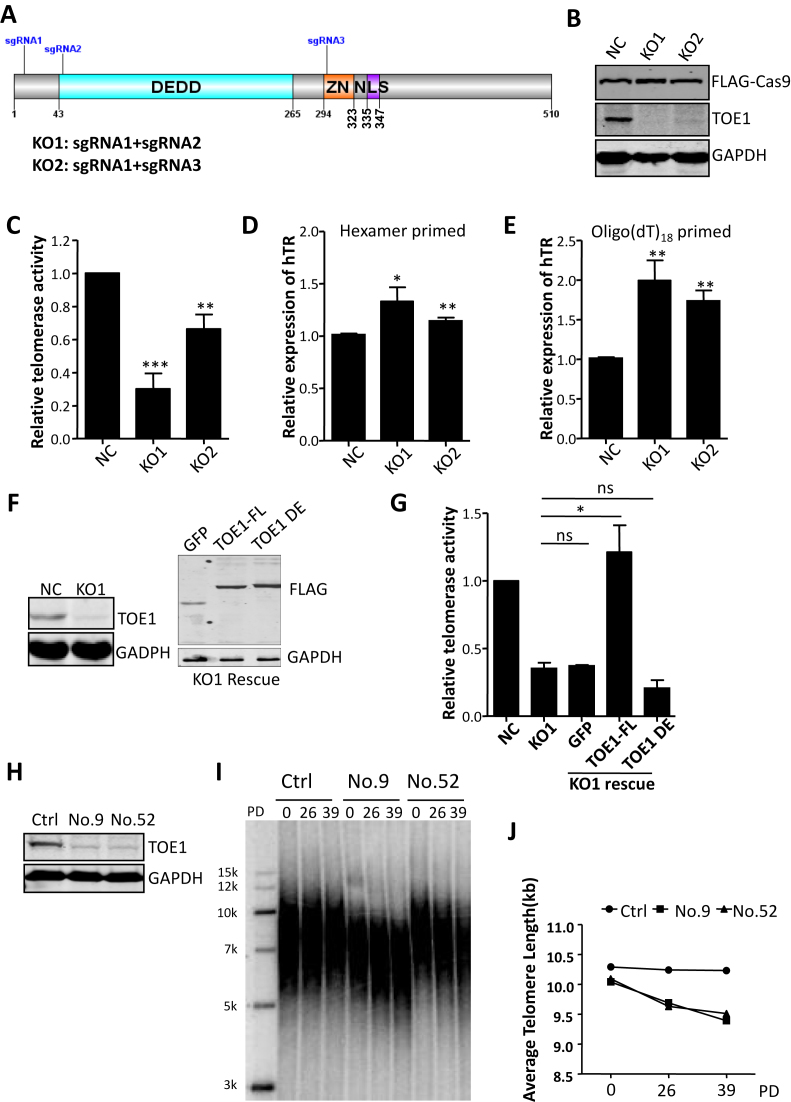
TOE1 is required for telomerase activity and telomere length maintenance. (**A**) Schematic representation of the dual gRNA targeting positions for knocking out TOE1. (**B**) Inducible TOE1 KO 293T cells were treated with doxycycline and KO efficiency was determined by western blot using the indicated antibodies. (**C**) The relative telomerase activity of cells from (B) was quantified by Q-TRAP. Error bars represent s.d. (*n* = 3, biological replicates). Significance was calculated using one-tailed unpaired *t*-test. **P < 0.01. (**D** and **E**) qRT-PCR analysis of hexamer-primed (D) or oligo(dT)-primed (E) hTR cDNAs prepared using RNA of cells from (B). Error bars represent s.d. (*n* = 3, biological replicates). *P < 0.05, **P < 0.01, one-tailed unpaired *t*-test. (**F**) Western blot analysis of TOE1 KO1 cells that stably expressed FLAG-HA-tagged GFP, TOE1-FL and TOE1-DE, using anti-FLAG, TOE1 and GAPDH antibodies. (**G**) The relative telomerase activity of control (NC), TOE1 KO (KO1) and TOE1 rescue cells (from F) were quantified by Q-TRAP. Error bars represent s.d. (*n* = 2, biological replicates). Significance was calculated using one-tailed unpaired *t*-test. *P < 0.05; ns, not significant. (**H**) Western blot analysis of TOE1 KO single clones No.9 and No.52, which were obtained from subcloning the incudible KO1 cells. (**I**) Cells from (H) were passaged over time and harvested at the indicated time points for TRF analysis. PD, population doubling. (**J**) Quantification of data from (I) using ImageJ software.

PARN deficiency can result in the accumulation of oligoadenylated hTR that is susceptible to exosome degradation, thus markedly reducing total hTR levels and giving rise to impaired telomerase activity and telomere shortening (24,54). These observations suggest that PARN mainly functions to remove oligo(A) tails. TOE1 was shown in previous studies to catalyze rapid deadenylation and slow 3′-to-5′ exonucleolytic processing of RNA substrates, which suggests that TOE1 is a processive 3′-to-5′ exonuclease and sets it apart from other DEDD-containing deadenylases (46). We reasoned that incomplete inhibition by siRNAs might have contributed to the moderate degree of decrease in telomerase activity in TOE1 KD cells, and therefore generated conditional TOE1 knockout (KO) 293T cell pools as previously described (57), in which Cas9 expression was controlled by a tetracycline-responsive promoter while TOE1-specific gRNAs were constitutively expressed (Figure 4A). Cells co-expressing inducible Cas9 and gRNAs against GFP were used as negative controls. Doxycycline addition led to robust Cas9 induction and efficient TOE1 deletion in cells expressing two different TOE1 gRNAs (Figure 4B). More importantly, a >70% decrease in telomerase activity could be observed, and the degree of reduction correlated with KO efficiencies of individual gRNAs (Figure 4C). Consistent with results from RNAi experiments, the percentage of hTR oligo(A) forms increased significantly in TOE1 KO cells, although we did not observe any decrease in total levels of hTR (Figure 4D and E). In fact, total amount of hTR increased slightly following TOE1 KO, possibly due to the accumulation of immature hTR precursors. Notably, ectopic expression of wild-type TOE1, but not the catalytically inactive TOE1-DE mutant, was able to fully rescue telomerase activities to control levels (Figure 4F and G). Similar results were also obtained using inducible TOE1 KO HeLa cells, which helps to exclude the possibility of cell type-specific responses (Supplementary Figure S3).

Given that reduced telomerase activity should lead to shortening of telomeres, we performed TRF assays to determine telomere length changes in the inducible TOE1 KO 293T line KO1. However, no telomere shortening was apparent in these cells (Supplementary Figure S4A). Upon closer examination, we discovered that TOE1 KO could suppress cell growth (Supplementary Figure S4B) and cells that still expressed TOE1 would dominate the cell population overtime (Supplementary Figure S4C). This finding was further supported by our failure to isolate any homozygous TOE1 KO single clones from these inducible cells. Consequently, we selected two heterozygous KO clones isolated from KO1 for TRF analysis (Figure 4H). In line with our findings of reduced telomerase activity upon TOE1 KD and KO, stable heterozygous TOE1 KO led to gradual and appreciable shortening of telomeres (Figure 4I and J; Supplementary Figure S4D and E). These results combined indicate that TOE1 is required for maintaining active telomerase and proper telomere length, likely through regulating hTR processing and maturation.

### TOE1 deficiency leads to the accumulation of 3′-extended and oligoadenylated hTR

**Figure 5.**
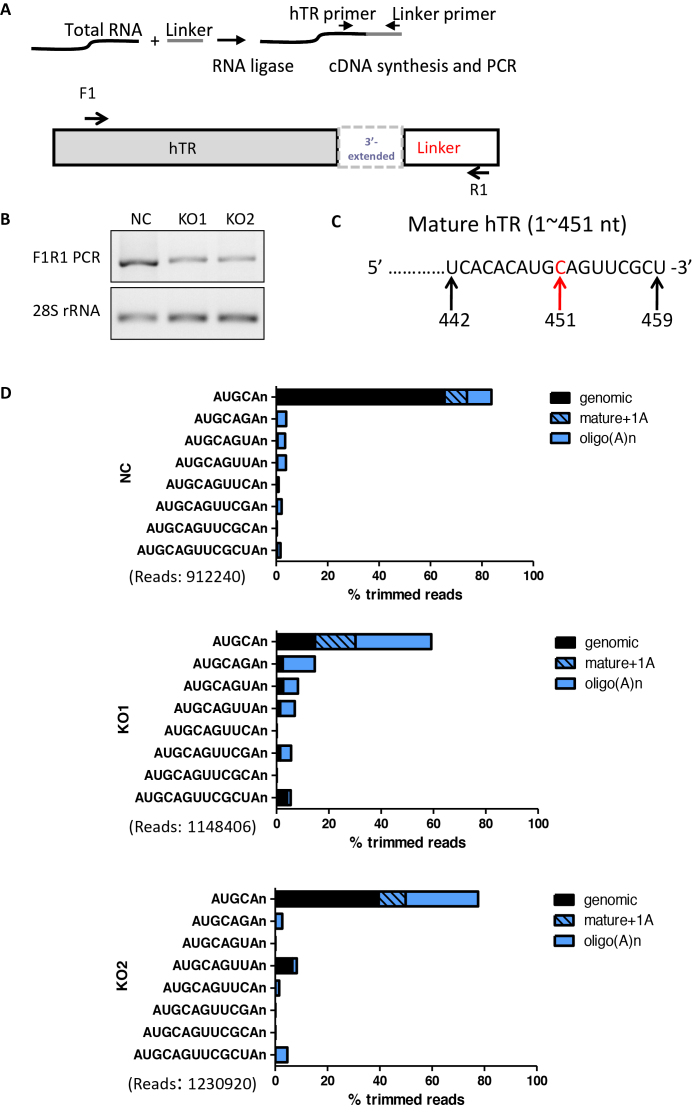
Accumulation of 3′-extended and oligoadenylated hTR in TOE1-deficient cells. (**A**) Strategy for hTR 3′ RACE. The primer set with F1 in the hTR and R1 in the linker was used to amplify hTR 3′ RACE products. (**B**) hTR 3′ RACE PCR products from control (NC) and inducible TOE1 KO HeLa cells (KO1 and KO2) (Supplementary Figure S3A) were resolved by agarose gel electrophoresis. (**C**) The 3′ end position 451 of the mature hTR (451 nt) is shown in red. (**D**) hTR 3′ RACE products from (B) were subjected to deep sequencing with reads aligned to the hTR gene. Genomically encoded termini are in black, mature hTR with a single adenosine (which may be genomically encoded) is hatched and oligo(A) additions (n≥2) are in solid blue. The total number of trimmed reads for each group is shown in parentheses.

While TOE1-deficient cells exhibited higher levels of oligo(A)-containing hTR and reduced telomerase activity, no corresponding decrease in total hTR amount was observed, suggesting TOE1 may be distinct from PARN (Figure 4D) (23). We speculated that TOE1-mediated 3′-end processing might affect hTR function more than its stability. To investigate this possibility more closely, we undertook 3′ RACE and high-throughput sequencing to monitor the 3′ ends of hTR in inducible TOE1 KO cells (Figure 5A). The slightly slower migrating PCR products from TOE1 KO cells hinted at the presence of longer forms of hTR (Figure 5B and C). Deep sequencing of these 3′ RACE products revealed a marked decrease in the proportion of mature hTR with the canonical end sequence of 5′-AUGC, especially for KO1, in line with more efficient TOE1 KO in KO1 cells (Figure 5D; Supplementary Figures S3A and 5). A diverse array of hTR variants with longer and non-canonical 3′ end sequences were upregulated with TOE1 KO, including those containing oligo(A) tails. In KO1 cells, a notable increase was observed for a hTR variant that contained a single adenosine addition at position 452 (Figure 5D and Supplementary Figure S5), a form that exhibited no change in PARN-deficient cells (23). Additionally, enrichment of nascent hTR transcripts with two to four bases beyond the canonical 3′ end (namely hTR with lengths of 453, 454 or 455 nt) was also noticeable. Similar changes in hTR variant distribution were observed in KO2 cells as well, albeit at a lower level, possibly due to less efficient TOE1 KO. Given that PARN remains intact in TOE1 KO cells, TOE1 likely functions non-redundantly with PARN.

We then undertook 3′ RACE and high-throughput sequencing to monitor the effect of TOE1 overexpression on hTR 3′ maturation using the same cells from Figure 3E (Supplementary Figure S6). Consistently, a marked decrease in the proportion of extended or oligoadenylated hTR was apparent, along with an increase in the proportion of mature hTR in wild-type TOE1 overexpressing cells, but not in cells expressing enzymatically dead TOE1 (Supplementary Figure S6). Taken together, our data support the notion that TOE1 participates in hTR 3′ end processing.

### TOE1 catalyzes deadenylation and 3′-to-5′ exonucleolytic trimming of hTR precursors

**Figure 6.**
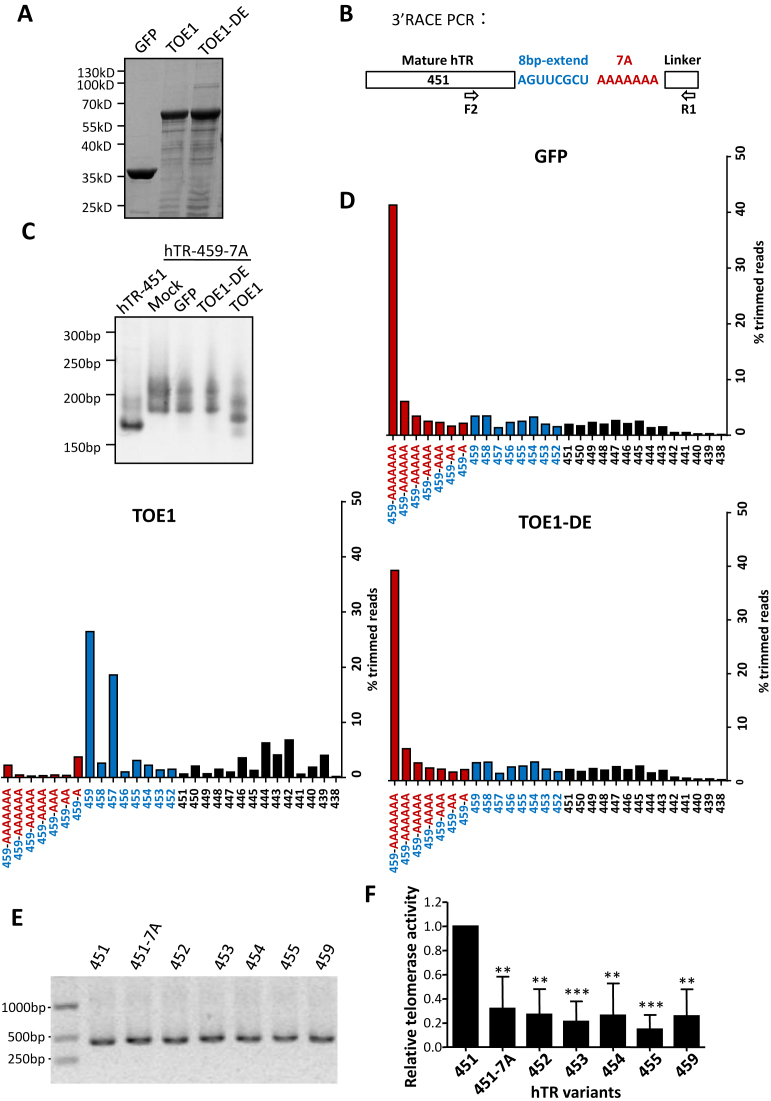
TOE1 can deadenylate hTR precursors and 3′-to-5′ exonucleotically process their 3′ end. (**A**) Bacterially purified His_6_-tagged wild-type and enzymatically dead (TOE1-DE) TOE1 were resolved by SDS-PAGE and visualized using Coomassie blue staining. His-tagged recombinant GFP was included as a negative control. (**B**) Schematic representation of the hTR precursor generated from *in vitro* transcription. The resulting substrate (hTR-459-7A) contains an 8-nt extension plus 7 adenosines. Forward primer F2 in the hTR region and reverse primer R1 in the linker region were used to amplify hTR 3′ RACE products. (**C**) The recombinant proteins from (A) (30 nM) were incubated with *in vitro* transcribed hTR precursors (60 nM) in an *in vitro* deadenylation assay. RNAs were then extracted for 3′RACE and the PCR products were resolved by PAGE. 3′RACE products using mature hTR (hTR-451) was included as a control. (**D**) The 3′RACE PCR products from (C) were subjected to deep sequencing. The percentages of trimmed reads were quantified and plotted as shown. (**E**) *In vitro* transcribed 3′-extended hTR PCR products were separated by agarose gel electrophoresis. The 451-7A variant contains 7 adenosines. (**F**) *In vitro* telomerase reconstruction assays were carried out using *in vitro* translated hTERT and *in vitro* transcribed hTR variants from (E). Activities of the reconstructed telomerase were assessed by Q-TRAP. Error bars represent s.d. (*n* = 3, biological replicates), **P < 0.01, ***P < 0.001, one-tailed unpaired *t*-test.

TOE1 and PARN appear to have redundant and distinct functions in RNA processing (58), likely a result of their being evolutionary paralogs (Supplementary Figure S7A). We next RNAi knocked down PARN in parental and inducible TOE1 KO HeLa cells (Supplementary Figure S7B and C). Consistent with published reports (23–24,54), PARN KD resulted in marked increase of oligoadenylated hTR (Supplementary Figure S7D and E). A similar accumulation of oligoadenylated hTR was also evident upon TOE1 KO, at ∼1/3 of the level of PARN KD cells (Supplementary Figure S7E). Notably, the accumulation of oligoadenylated hTR was augmented in PARN/TOE1 co-depleted cells. These results support functional similarities between TOE1 and PARN as a deadenylase for hTR and suggest that TOE1 and PARN are not interchangeable. To better probe the mechanism of TOE1 modulation of hTR, we next performed *in vitro* deadenylation assays using bacterially purified His-tagged wild-type and catalytically dead TOE1 (Figure 6A). As substrates, we *in vitro* transcribed an extended hTR precursor that contained 8 additional nucleotides beyond position 451 plus a 7-adenosine tail (hTR-459-7A) (Figure 6B). *In vitro* processed RNAs were then extracted for 3′ RACE PCR analysis, the products of which were resolved by PAGE (Figure 6C). As expected, wild-type TOE1 but not enzymatically dead TOE1 (TOE1-DE) was able to process hTR-459-7A, as evidenced by the presence of faster migrating species in reactions with wild-type TOE1 (Figure 6C, lane 5). The 3′ RACE products were then deep sequenced (Figure 6D), which revealed a prevalence of products that ended at positions 459 and 457. This result implied a switch from deadenylation to exonucleolytic digestion at these positions by TOE1, which is consistent with the hypothesis of TOE1 being a 3′-to-5′ exonuclease that processes hTR 3′ end after deadenylation. Previous reports suggest that TOE1 catalyzes rapid deadenylation (46). However, in contrast to other known deadenylases, TOE1 also harbors a much slower 3′-to-5′ exonucleolytic activity (46), suggestive of less efficient cleavage of non-poly(A) sequences by TOE1. Our results support a role of TOE1 in modulating the 3′ maturation of hTR as a deadenylase and a 3′-to-5′ exonuclease of hTR precursors.

We reasoned that knocking out TOE1 would lead to the accumulation of improperly processed hTR variants, whose incorporation into the telomerase complex (not changes in total hTR levels *per se*) might account for the observed decease in telomerase activities in TOE1 KO cells. To explore this possibility, we carried out telomerase reconstitution assays using *in vitro* translated hTERT proteins and *in vitro* transcribed hTR variants, including hTR 451-7A that contained seven additional adenosines (Figure 6E). Compared to ‘wild-type’ hTR 451, *in vitro* assembly of telomerase complexes using all of the 3′-extended hTR forms yielded significantly diminished activities (Figure 6F), supporting the notion that these improperly processed hTR products are defective in reconstituting telomerase activities.

## DISCUSSION

To date, both the mechanisms by which primary hTR transcripts are processed into mature hTR and the enzymes involved in the multi-step process remain poorly understood. The discovery of IPF and DC patients with mutations in PARN has offered crucial clues (25,26). In these patients, both hTR processing and telomerase activity are affected (23). We found that TOE1 deficiency led to the accumulation of 3′-extended and polyadenylated hTR precursors and decreased the ratio of mature hTR, resulting in impaired telomerase activity and shortened telomeres. While these findings underscore the similarities between TOE1 and PARN, our results also highlight the non-redundant role that TOE1 plays in hTR regulation. Previously, PARN was found to catalyze deadenylation reactions at a concentration of 0.1125 nM (59), while TOE1 worked at 10–50 nM (46), which is indicative of TOE1’s weaker deadenylase activity compared to PARN. TOE1 was also reported to carry out a slower 3′-to-5′ exonucleolytic activity following an initial faster deadenylation reaction (46), suggesting that TOE1 cleaves non-A nucleotides less efficiently. These observations point to distinct catalytic properties between TOE1 and PARN.

PARN is localized in both the nucleolus and CB. 3′-extended hTR transcripts primarily reside in the nucleolus where the human TRAMP complex and exosome subunits are also found (24,60–61). In comparison, TOE1 is specifically targeted to CBs, where telomerase processing and assembly takes place (24,34,41,61). Moreover, hTR’s nucleolar 3′-end processing is thought to occur prior to its translocation to CBs (62). We therefore speculate that the 3′ ends of hTR may be processed by multiple exonucleases, with spatial and temporal dependence. Two such enzymes are PARN and TOE1, which may act on hTR sequentially or simultaneously.

**Figure 7.**
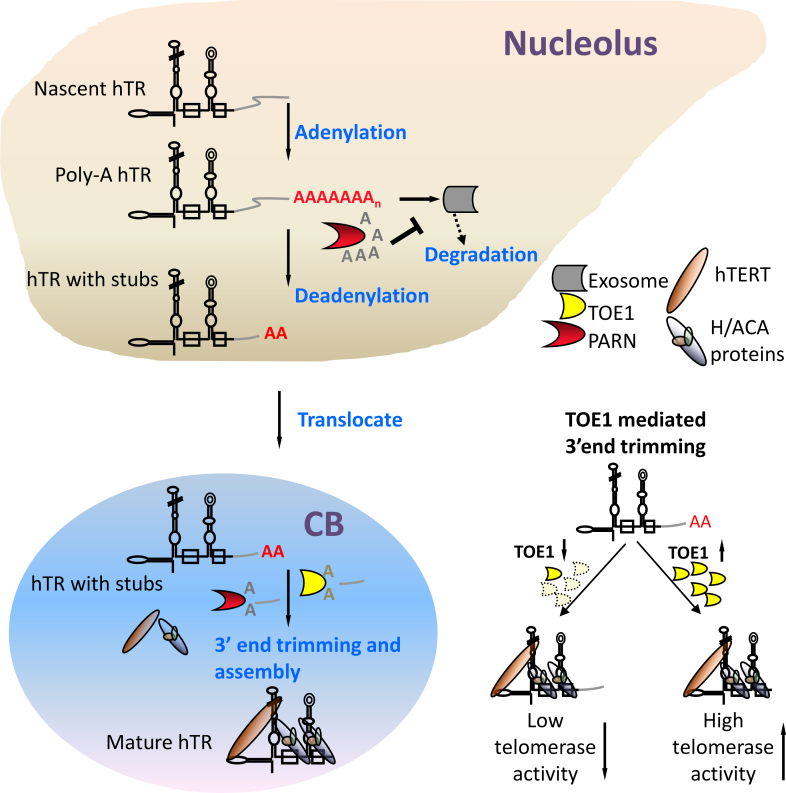
A Model for TOE1-mediated telomere maintenance through its exonucleolytic processing of telomerase RNA hTR. Nascent hTR transcripts contain genomically encoded 3′-extensions beyond the mature 451 nt (black). Termination of hTR transcription is coupled to oligo-adenylation, which targets transcripts for degradation by the exosome. PARN counteracts the degradation pathway by removing oligo(A) tails in nucleoli. After deadenylation, near mature versions of hTR that contain genome-encoded 3′-extended stubs and shorter non-templated 3′ adenosine tails transit from nucleoli to CBs for further processing by PARN and TOE1. TOE1 catalyzes 3′-to-5′ exonucleolytic decay of hTR precursors and trims genomically encoded bases (gray) of nascent hTR to yield mature 3′-ends. If TOE1 is deficient, hTR precursors with 3′-extended tails accumulate and may be incorporated into the telomerase complex, resulting in lower telomerase activity.

In our model (Figure 7), PARN competes with the exosome complex and trims the 3′ ends of hTR precursors in nucleoli. This trimming process represents one of the first maturation steps of hTR by removing most poly(A) tails. The resultant near mature versions of hTR may contain genome-encoded 3′-extended stubs and shorter non-templated 3′ adenosine tails. These processed hTR precursors may transit from nucleoli to CBs for further processing. In CBs, the remaining oligo(A) tail is removed prior to 3′-extended stubs by TOE1 and/or PARN, cooperatively or sequentially. In *in vitro* deandeylation assays, PARN catalyzed both deadenylation and 3′-to-5′ exonucleolytic decay faster than TOE1, removal of 3′-extended stubs by PARN is therefore more efficient. It is possible that longer 3′-extended stubs are trimmed by PARN, while shorter stubs, especially those closer to position 451, may be more precisely processed by TOE1. Consistent with this idea, 3′ RACE sequencing revealed an increase of hTR transcripts with 453, 454 and 455 nt in TOE1 KO1 cells (Figure 5D). When TOE1 is deficient, a small proportion of 3′-extended hTR with oligo(A) tails may accumulate in CBs, resulting in more immature hTR precursors but no decrease in total hTR levels. And the incorporation of these improperly extended hTR variants into the telomerase complex leads to decreased telomerase activities.

PARN can form homodimers (63). We found that TOE1 could also bind to itself in co-IP experiments (Figure 1B). Whether homodimerization is important to their activities or functions is unclear. PARN was also found in the TOE1 protein complex in previous IP-MS studies (33), but any possible heterodimerization has yet to be tested. More work is warranted to identify the compartments in which different hTR precursors localize, the various enzymes that modify hTR, and the interplay between different hTR processing pathways. Such studies will not only help to elucidate how disruption of hTR processing and the incorporation of improperly processed hTR may affect telomerase stability and activity, but also facilitate our understanding of the development and pathogenesis of diseases such as PCH7 and DC.

## DATA AVAILABILITY

The original data have been submitted to SRA database with accession number SRP145157.

## Supplementary Material

Supplementary Data
