# Supplementary material for: TOE1 acts as a 3′ exonuclease for telomerase RNA and regulates telomere maintenance
Source: Nucleic Acids Res. 2018 Oct 29;47(1):391–405. doi: 10.1093/nar/gky1019 (PMC6326811; doi:10.1093/nar/gky1019)
Supplement: Supplementary Data [file gky1019_supplemental_files.pdf]

S1 : supplemental data to Figure 1

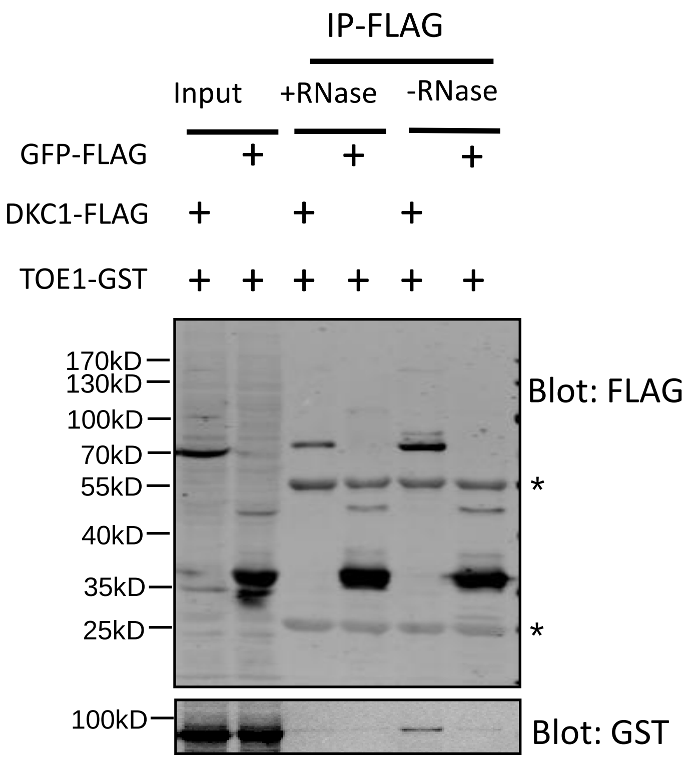

Figure S1. Western analysis of whole-cell extracts (Input) and Flag immunoprecipitates (IP) with or without RNase treatment prepared from 293T cells that transiently express Flag-HA -tagged GFP and DKC1 together with GST-TOE1 using Flag and GST antibodies respectively. Asterisks indicate the positions of denatured heavy and light chains.

S2 : supplemental data to Figure 3

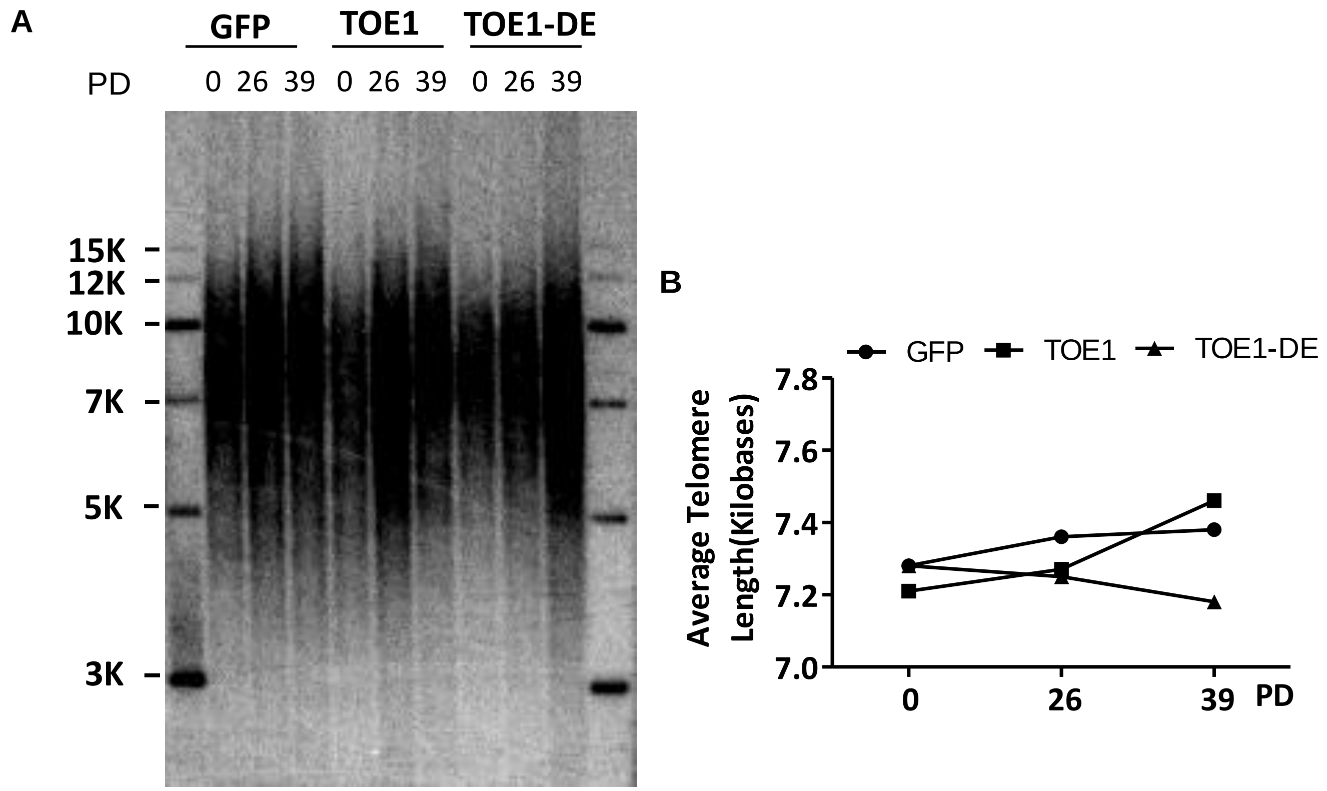

Figure S2. Telomere length measurement in TOE1 overexpressed 293T cells during passages (biological repeat for Fig 3I/J). (a). 293T cells that stably expressed FLAG-HA-tagged GFP, TOE1-FL and TOE1-DE were harvested at the indicated time points. Telomere restriction fragment (TRF) analysis of cells from the indicated PDs was performed to determine average telomere length. PD, population doubling. (b) Quantification of data from (a) using ImageJ software.

S3 : supplemental data to Figure 4

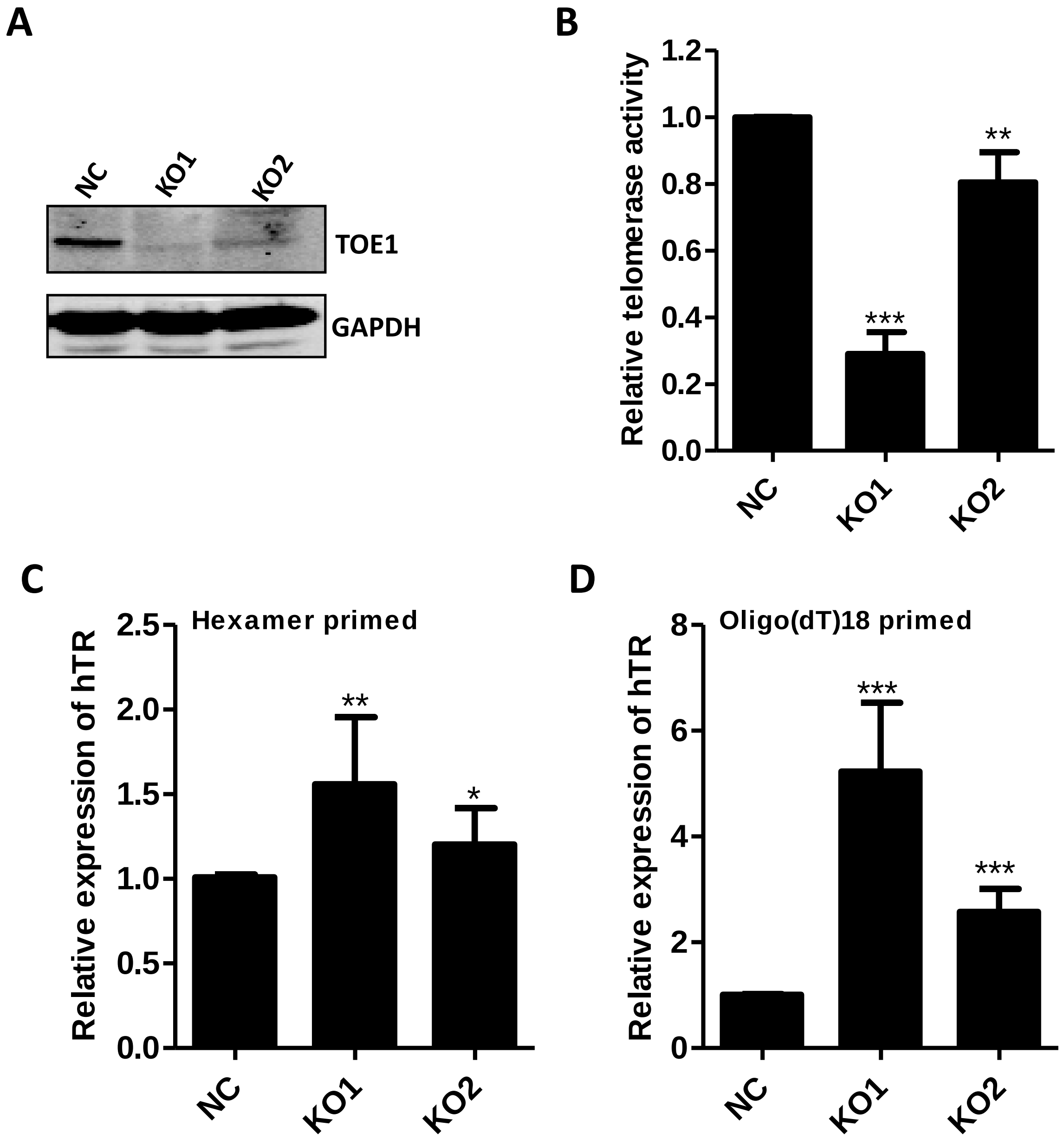

Figure S3. Inducible knockout TOE1 in Hela cells decreased telomerase activity and accumulated levels of hTR precusors with oligo(A). (a).Inducible knockout TOE1 with doxycycline in Hela cells and the knockout efficiency was checked by western blot using TOE1 and GAPDH antibodies. (b). The telomerase activity of whole cell lysate from (a) was quantified by Q-TRAP assay. (c and d). qRT-PCR analysis of hTR from hexamer-primed or oligo(dT)-primed cDNA prepared using RNA from (a). n=3, biological replicates. Error bars represent s.d., *$P < 0.05$, ** $P < 0.01$, *** $P < 0.001$, one-tailed unpaired *t*-test.

S4 : supplemental data to Figure 4

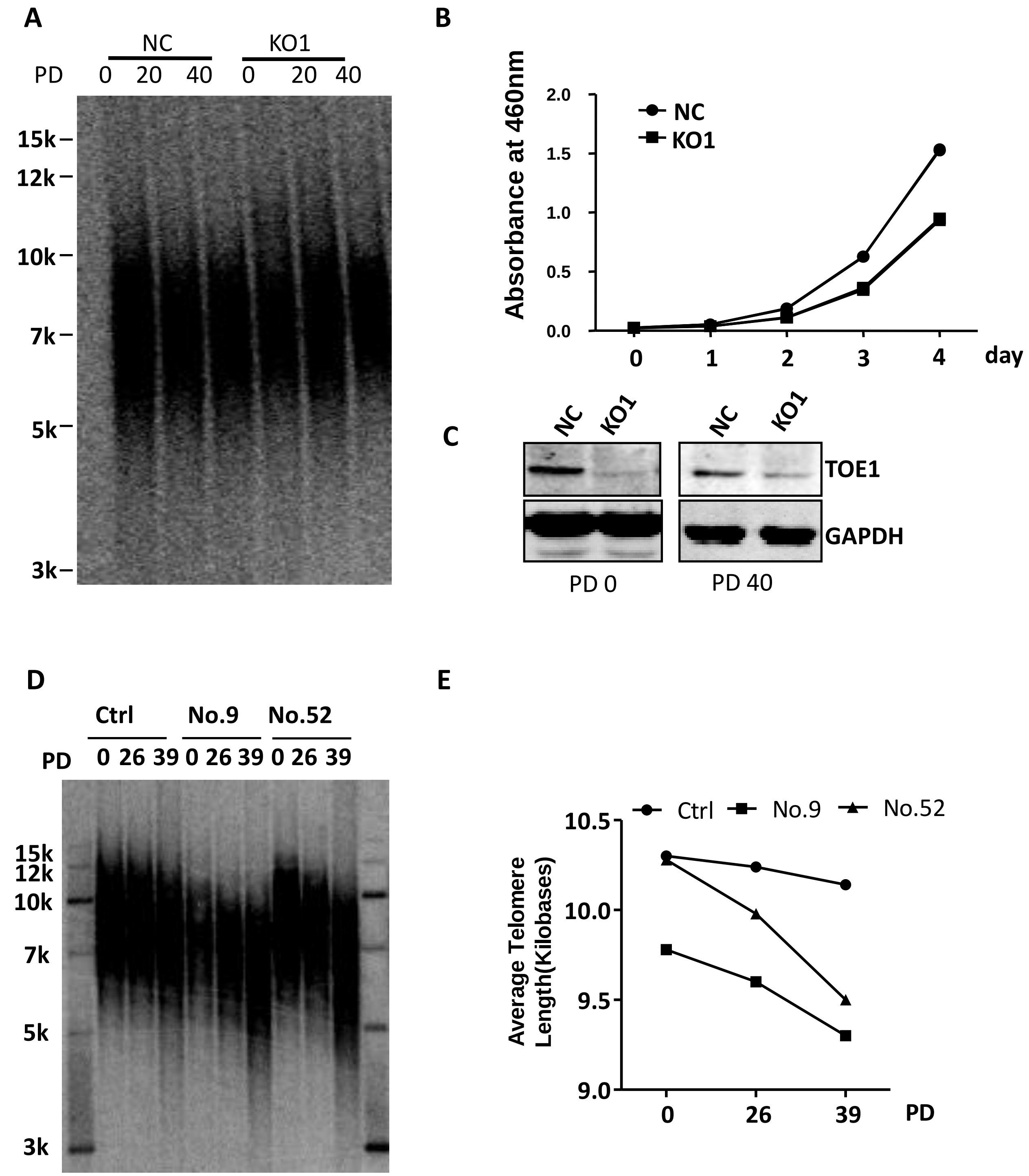

Figure S4. Telomere length measurement, growth curve and knockout efficiency of TOE1 KO cells during passages. (a). TOE1 KO1 or negative control （NC）of 293T cells were generated and passaged over time. Cells were harvest at the indicated time points. Telomere restriction fragment (TRF) analysis of cells from indicated population doublings (PDs) was performed to determine average telomere length. (b).Cell growth curve of TOE1 KO1 and NC of 293T cells were performed and calculated by using Cell Counting Kit-8 (CCK-8). (c). Western blot analysis of whole-cell extracts from PD0 and PD40 of TOE1 KO1 or NC of 293T cells using TOE1 and GAPDH antibodies. (d-e). Biological repeat for Fig 4I/J. 293T TOE1 KO single clone NO.9 and NO.52 were harvested at the indicated time points. Telomere restriction fragment (TRF) analysis of cells from the indicated PDs was performed to determine average telomere length. PD, population doubling (d). TRF length was calculated using ImageJ software (e).

S5 : supplemental data to Figure 5

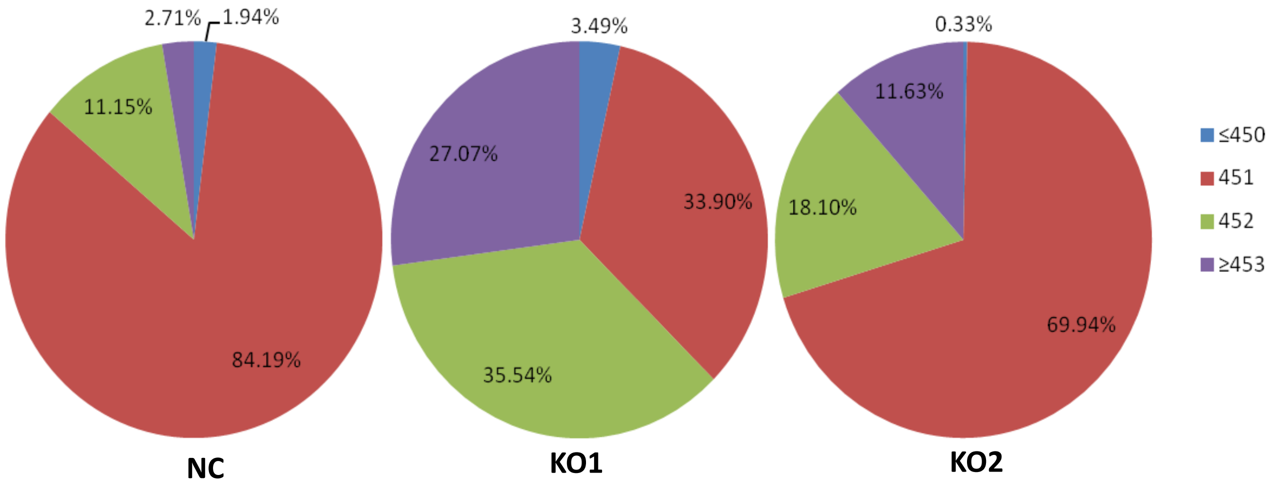

Species with genomically encoded termini

Figure S5. Pie chart illustrating distribution of the 3’ end position of genomically encoded termini of hTR from 3’RACE hTR products. 3’-truncated (≤450), mature (451), 452, and longer (≥453) forms of hTR are shown in different colors.

S6: supplemental data to Figure 5

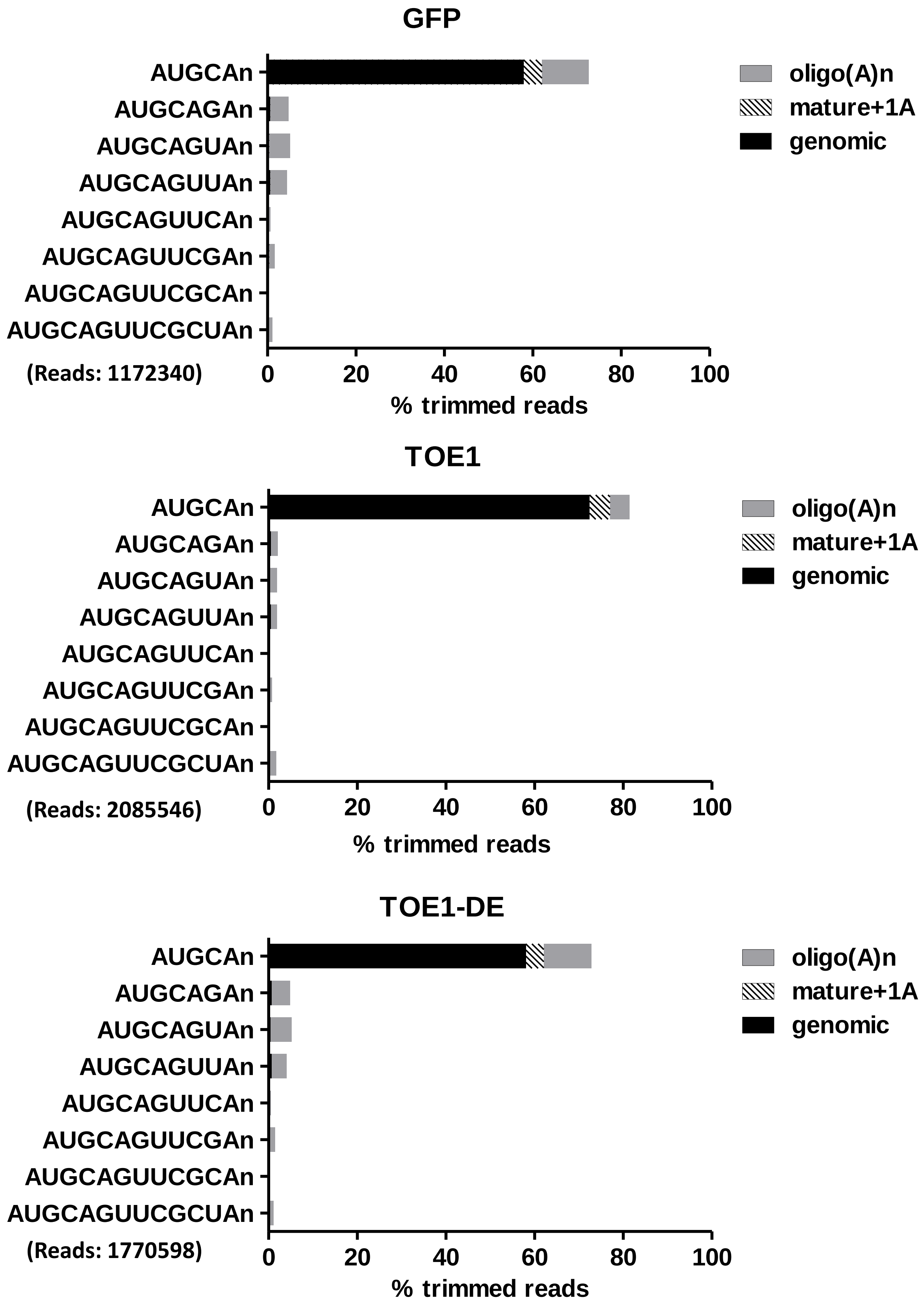

Figure S6. hTR 3' RACE PCR products from 293T cells overexpressing GFP/TOE1/TOE1-DE mutant (Figure 6) were subjected to deep sequencing, and reads were aligned to the hTR gene. Genomically encoded termini are in black, mature hTR with a single adenosine (which may be genomically encoded) is hatched and oligo(A) additions of any length (n≥2) are in grey.

S7: supplemental data to Figure 6

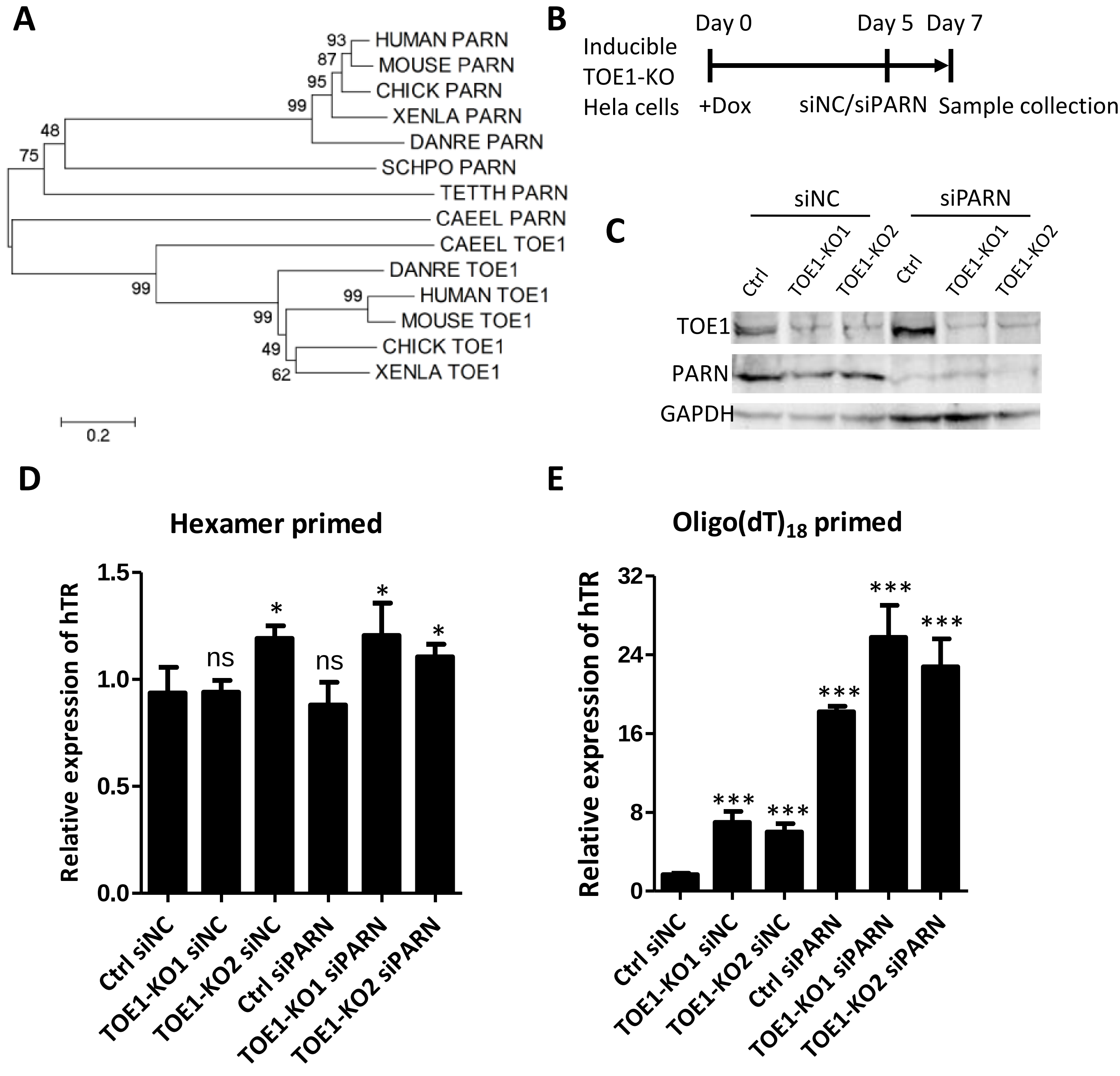

Figure S7. 3'-maturation of telomerase hTR is regulated by both TOE1 and PARN. (a). A phylogenetic tree of TOE1 and PARN in multiple species. HUMAN: Homo Sapiens; MOUSE: Mus musculus; CHICK: Gullus gallus; XENLA: Xenopus Laevis; DANRE: Danio rerio; SCHPO: Schizosaccharomyces pombe; TETTH: Tetrahymena thermophila; CAEEL: Caenorhabditis elegans. (b). Strategy of knocking down PARN in inducible TOE1 KO HeLa cells. (c). Western blot analysis of TOE1 and PARN protein levels after single or double loss of function cells. (d, e). qRT-PCR analysis of hTR RNA from hexamer primed (d) or oligo(dT)-primed (e) cDNA from (c). Error bars represent s.d. (n=3, technical replicates), *P < 0.05, **P < 0.01, ***P < 0.001, ns, not significant, one-tailed unpaired *t*-test.
